# Validity Evaluation Method Based on Data Driving for On-Line Monitoring Data of Transformer under DC-Bias

**DOI:** 10.3390/s20154321

**Published:** 2020-08-03

**Authors:** Yuanda He, Qi Zhou, Sheng Lin, Liping Zhao

**Affiliations:** School of Electrical Engineering, Southwest Jiaotong University, Chengdu 611756, China; he_yuanda@my.swjtu.edu.cn (Y.H.); zqi@my.swjtu.edu.cn (Q.Z.); lpzhao@swjtu.cn (L.Z.)

**Keywords:** transformer, DC-bias, on-line monitoring, data validity evaluation, data driving

## Abstract

The DC-bias monitoring device of a transformer is easily affected by external noise interference, equipment aging, and communication failure, which makes it difficult to guarantee the validity of monitoring data and causes great problems for future data analysis. For this reason, this paper proposes a validity evaluation method based on data driving for the on-line monitoring data of a transformer under DC-bias. First, the variation rule and threshold range of monitoring data for neutral point DC, vibration, and noise of the transformer under different working conditions are obtained through statistical analysis. Then, the data validity criterion of DC bias monitoring data is proposed to achieve a comprehensive evaluation of data validity based on data threshold, continuity, impact, and correlation. In addition, case studies are carried out on the real measured data of the DC bias magnetic monitoring system of a regional power grid by using this evaluation method. The results show that the proposed method can systematically and comprehensively evaluate the validity of the DC bias monitoring data and can judge whether the monitoring device fails to a certain extent.

## 1. Introduction

The main transformer of the urban power grid is affected by the stray current of the subway, which produces the phenomenon of DC bias [1,2,3]. The concrete performance of DC bias is that the vibration of the transformer intensifies and the noise increases [4]. Severe DC bias will affect the working life of the transformer and even cause permanent damage to the transformer [5,6]. In view of this, on-line monitoring devices for DC bias have been installed in many urban power grids in China, such as Shanghai, Guangzhou, Guiyang, and so on [7,8]. The installation of the DC bias monitoring device realizes the on-line monitoring of neutral point DC, vibration, and noise of the transformer [9,10].

However, in the practical application, the DC bias monitoring sensors are easily affected by external noise interference, equipment aging, and communication failure [11,12,13]. Therefore, it is difficult to guarantee the validity of DC bias monitoring data which causes problems for the DC bias state judgment and characteristic analysis of the main transformer. For example, the audible noise measurement sensor is often interfered with by background noise [11]. Most monitoring devices are installed outdoors, where the operating environment is harsh. And the service life of monitoring devices is far lower than that of the power transformer itself [12]. In addition, there are problems of unreliability in the process of remote monitoring data transmission [13]. Therefore, to solve the above problems, it is of great engineering application value to study the validity evaluation method for on-line monitoring data of the transformer under DC-bias.

At present, some research has been carried out to solve the problem that the validity of DC bias monitoring data is difficult to guarantee in the practical application [13,14,15]. However, the existing studies can only improve the validity of DC bias monitoring data partly. For example, a method has been proposed to solve the problem of unreliability in remote data transmission based on communication fault detection [13]. Liu et al. [14] and Tong et al. [15] proposed new monitoring methods to solve the overload and anti-interference problems of neutral DC monitoring sensors, respectively. To the best of our knowledge, there is no method to comprehensively solve the problem of poor validity of monitoring data in the field of DC bias monitoring.

However, in other fields, scholars have conducted many studies on the evaluation method which can solve the problem of poor validity of monitoring data [16,17,18]. Because the valid data and invalid data can be clearly distinguished by using the data validity evaluation method, through analyzing the causes of invalid data, the problems existing in the monitoring device can be solved. However, the existing data validity evaluation methods are target specific data, such as land change data [17] and urban traffic data [18], and the types and variation characteristics of transformer DC bias monitoring data are quite different from the above monitoring data. Therefore, it is necessary to propose a method focused on the validity evaluation of DC bias monitoring data.

In addition, data-driven methods have been widely applied in the fields of status monitoring [19], anomaly detection [20], and residual life estimation [21]. Unlike the traditional methods based on physics models, data-driven methods do not need to know the specific information of the objects’ mathematical model [22], and data-driven methods can control and evaluate the system only requiring the monitoring data [23,24].

The objective of this paper is to solve the problem that the validity of DC bias monitoring data is difficult to guarantee in practical application. Thus, a data-driven and multi-criterion method is proposed to achieve the validity evaluation of DC bias monitoring data in this paper. On the one hand, this paper evaluates the validity of monitoring data by using the data-driven method, which does not need to know the specific information of the transformer model and avoids the complex electromagnetic analysis of transformer. On the other hand, the multi-criterion method proposed in this paper considering data threshold, continuity, impact, and correlation is more comprehensive than the single-criterion method in the evaluation process, which can cover as many abnormal cases as possible. Finally, case studies have been carried out to verify the correctness of the proposed method in this paper.

The remainder of this paper is organized as follows. The transformer DC bias monitoring devices and characteristics of normal and abnormal monitoring data are presented in Section 2. Section 3 presents the data validity criteria and evaluation process. The results of the case studies are presented in Section 4. Section 5 concludes the whole paper.

## 2. Data Characteristics Analysis

### 2.1. Transformer DC Bias Monitoring Device

When the DC bias of the main transformer occurs, the neutral point DC of the main transformer increases, the vibration intensifies, and the abnormal noise is obvious [7]. Therefore, to accurately identify DC bias hidden danger, the transformer DC bias synchronous monitoring device based on neutral point DC, vibration, and noise is widely used [8,9].

**Figure 1:**
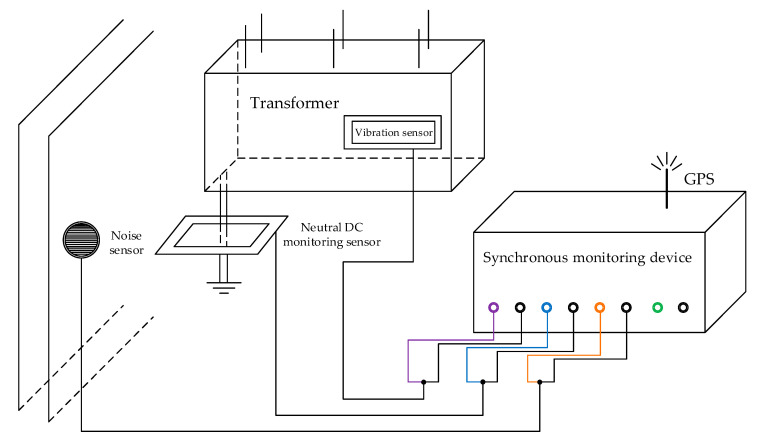
The composition and connection mode of the monitoring system.

**Figure 2:**
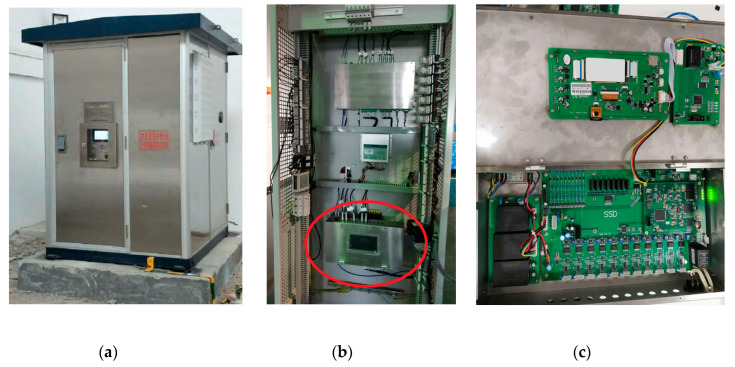
The hardware of the DC bias monitoring device of the transformers: (**a**) device appearance; (**b**) synchronous monitoring device; (**c**) physical picture of internal hardware.

Take the transformer DC bias monitoring system of a certain area power grid as an example. The composition and connection mode of the monitoring system are shown in Figure 1, and the hardware of the DC bias synchronous monitoring device of the transformers is shown in Figure 2. The DC bias monitoring system of the transformer is mainly composed of a synchronous monitoring device, neutral DC monitoring sensor, vibration speed sensor, and noise sensor. The synchronous monitoring device collects and displays real-time data from the other three sensors. At the same time, real-time synchronous data will be transmitted to the background monitoring system through the global position system (GPS).

**Figure 3:**
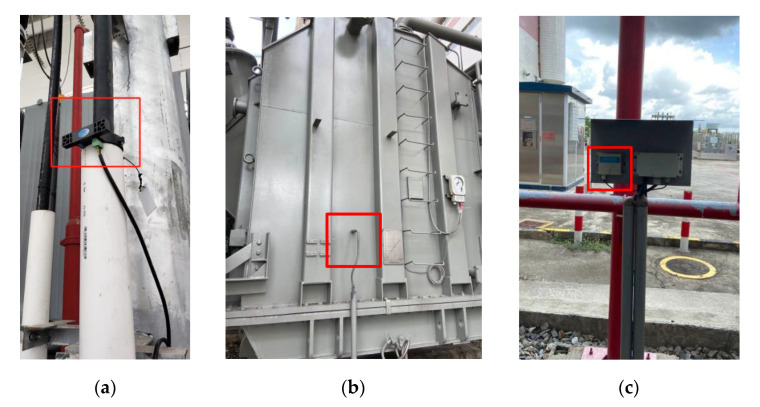
The installation mode of sensors: (**a**) neutral DC monitoring sensor; (**b**) vibration sensor; (**c**) noise sensor.

The installation mode of the neutral DC monitoring sensor, vibration sensor, and noise sensor is shown in Figure 3. The objects of the monitoring device are 220 kV and 500 kV transformers in the power grid. The basic information of the main transformer being monitored is shown in Table 1. Each transformer is provided with a set of monitoring devices including three sensors.

### 2.2. Characteristics of Normal Monitoring Data

In this part, the characteristics of neutral point DC, vibration, and noise monitoring data of the transformer under different working conditions are analyzed, which lays a foundation for obtaining the criterion of data validity evaluation.

#### 2.2.1. The Classification of Working Conditions

**Figure 4:**
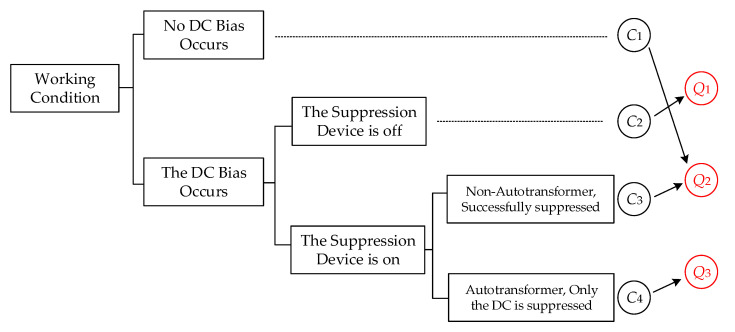
The classification of working conditions.

To distinguish the characteristics of normal data under different working conditions, the specific classification method is shown in Figure 4. Firstly, according to the occurrence of DC bias, the working conditions were divided into two categories. Secondly, according to whether the DC bias suppression device was turned on, the secondary classification was carried out. When DC bias did not occur, whether the suppression device was opened or not had no effect on the monitoring data. Therefore, a secondary classification of this working condition was not made. Thirdly, the third classification was made according to whether the transformer was an autotransformer. Similarly, it did not matter whether the transformer was an autotransformer or not when the suppression device was off. Therefore, a third classification of this working condition was not made. To sum up, four working conditions were obtained, *C*_1_–*C*_4_.

#### 2.2.2. Data Characteristics without DC Bias

**Figure 5:**
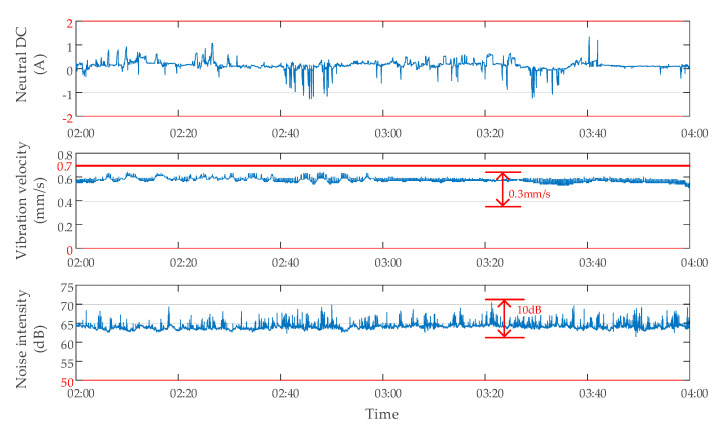
Data characteristics of a transformer without DC bias.

**Figure 6:**
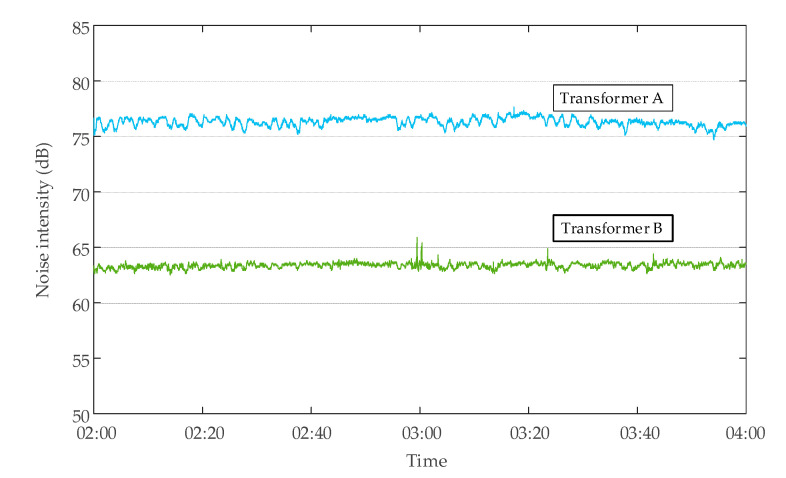
The noise intensity of transformer A and transformer B in the period from 2:00 to 4:00.

To obtain the data characteristics of the transformer without DC bias, the tests were carried out. There was no subway stray current interference at 02:00–04:00 at night, and no DC bias occurred to the transformer. The monitoring data of neutral point DC, vibration, and noise of a transformer are shown in Figure 5. The neutral point DC changed within ±2 A. The vibration velocity was within 0 and 0.7 mm/s, and the amplitude of change did not exceed 0.3 mm/s. The noise intensity was within 50 and 90 dB, and the amplitude of change did not exceed 10 dB. In addition, the average noise intensity of different transformers without DC bias was different. As shown in Figure 6, the average noise intensity of transformer A during the period of 02:00–04:00 was 76.30 dB, while the average noise intensity of transformer B was only 63.36 dB.

#### 2.2.3. Data Characteristics When the DC Bias Occurs and the Suppression Device Is off

**Figure 7:**
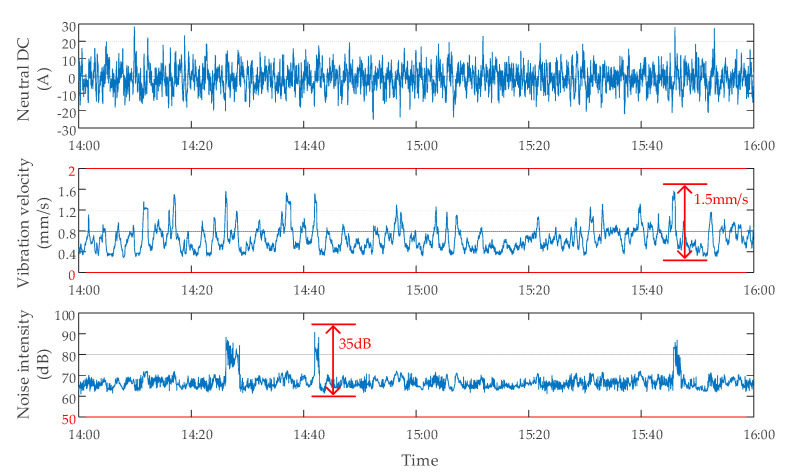
The data characteristics of a transformer with DC bias and suppression device off.

When DC bias of transformer occurred, the use of the DC bias suppression device had a great influence on the monitoring data [25,26,27]. To obtain the data characteristics when the DC bias occurred and the suppression device was off, the tests were carried out. During the operation of the subway from 14:00 to 16:00, DC bias of the transformer occurred, and the suppression device was off. The monitoring data of neutral point DC, vibration, and noise of a transformer are shown in Figure 7. The neutral point DC fluctuated within ±90 A. Vibration velocity was within 0 and 2 mm/s, and the maximum variation amplitude did not exceed 1.5 mm/s. The noise intensity was within 50 and 110 dB, and the maximum variation range did not exceed 35 dB.

#### 2.2.4. Data Characteristics When the DC Bias Occurs and the Suppression Device Is on

**Figure 8:**
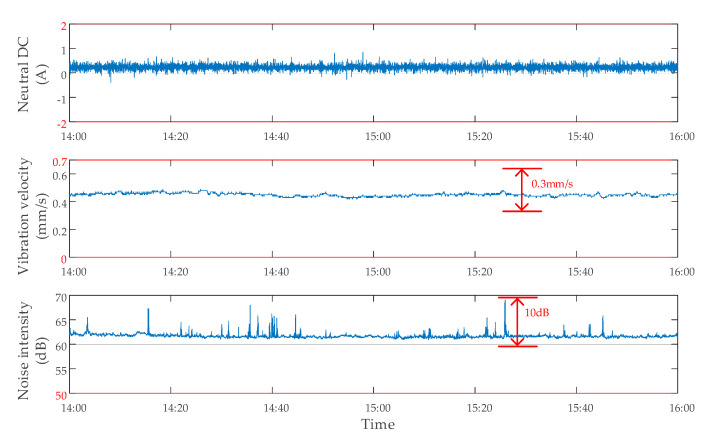
The data characteristics of a transformer with DC bias and suppression device on.

**Figure 9:**
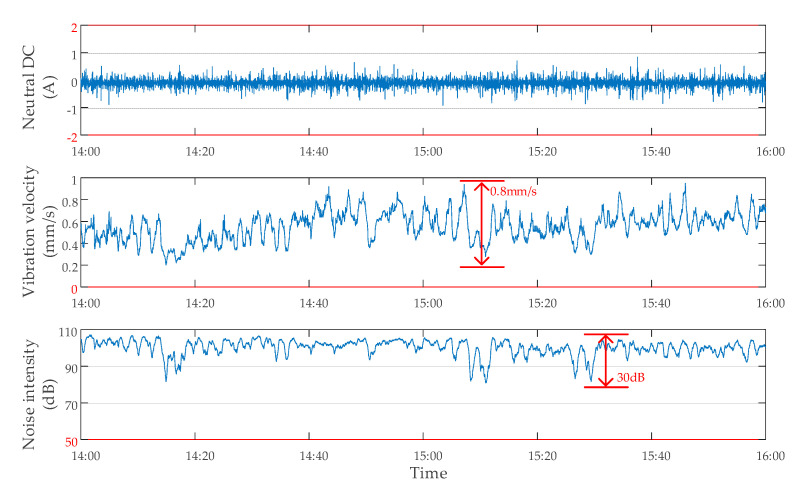
Data characteristics of an autotransformer when suppression device is on but fails to suppress DC bias.

To obtain the data characteristics when the DC bias occurs and the suppression device is on, the tests were carried out. During the operation of the subway from 14:00 to 16:00, DC bias of the transformer occurred, and the suppression device was on. The monitoring data of neutral point DC, vibration, and noise of a transformer are shown in Figure 8. The data characteristics are like those without DC bias. In addition, considering the particularity of the autotransformer, the DC bias of the autotransformer cannot be effectively suppressed by adding a single suppression device [28]. In this case, the neutral point DC varied within ±2 A, indicating that DC was indeed limited. However, the vibration velocity varied within 0 and 2 mm/s, and the noise intensity varied within 50 and 110 dB, indicating that DC bias was not successfully suppressed, as shown in Figure 9.

#### 2.2.5. Summary of Characteristics of Normal Monitoring Data

By comparing the characteristics of the above transformer DC bias monitoring data, it can be seen that the data characteristics of working conditions *C*_1_ and *C*_3_ were the same. Therefore, we classified the two conditions into the same class. The final classification result, *Q*_1_–*Q*_3_, is shown in Figure 4. The first type *Q*_1_ was that the transformer had DC-bias and the suppression device was off. The second type *Q*_2_ consisted of two cases: the transformer had no DC-bias, the non-autotransformer had DC-bias, and the suppression device was on. The third type *Q*_3_ was that the autotransformer had DC-bias and the suppression device was on. The data characteristic rules under different working conditions are shown in Table 2.

### 2.3. Characteristics of Abnormal Monitoring Data

Abnormal data often correspond to various fault situations; therefore, the evaluation of data validity can be completed by analyzing the characteristics of abnormal data caused by various fault situations. Abnormal data types [29,30,31] are mainly:

1. Blank data: the monitoring value is always empty or zero. It is caused by the inductive damage of the monitoring device or the interruption of system transmission.
2. Over range data: the monitoring value exceeds the allowable measurement range of the sensor. It is caused by the strong external interference to the sensor or the system communication failure.
3. Offset data: there is a certain deviation between the monitored value and the real value. It is caused by the aging of the sensor detection unit.
4. Abnormal zero drift data: the monitoring value is deviated abnormally with the change of time. It is caused by the aging of the sensor.
5. Variable ratio deviation data: there is a certain proportion relationship between the monitored value and the real value. It is caused by the change of the external environment or the misconfiguration of the sensor.
6. Abnormal mutation data: abnormal mutation of the monitoring value. It is caused by the strong external interference to the sensor or the failure of the sensor itself.
7. Abnormal step data: the monitoring value has an unreasonable and sudden change. It is caused by the change of the external environment or the strong interference to the sensor.

**Figure 10:**
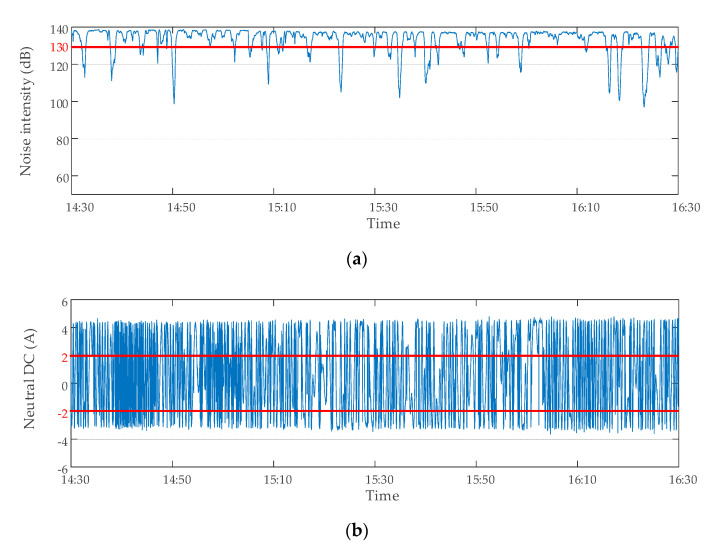

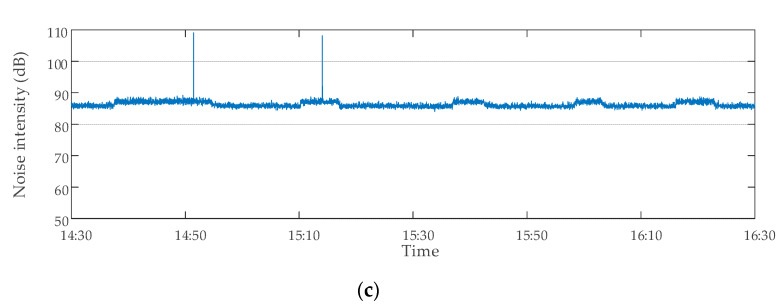
Several typical abnormal data: (**a**) noise overrange data caused by abnormal migration; (**b**) abnormal zero drift data of neutral DC; (**c**) noise abnormal mutation data.

Through the analysis of the existing data, it can be found that the above seven kinds of abnormal data of DC bias monitoring do not occur in isolation, but often occur at the same time. Therefore, it was necessary to analyze the data with multiple criteria at the same time, so as to evaluate the validity of the data more accurately. Several typical abnormal data are shown in Figure 10.

## 3. Methods of Data Validity Evaluation

In this section, according to the characteristics of normal data and abnormal data, this paper proposes data validity criteria based on data threshold, continuity, impact, and correlation. Each criterion reflects the validity of the data in one respect. Therefore, a method for evaluating the validity of data with multiple criteria is presented in this paper.

### 3.1. Data Validity Criterion

#### 3.1.1. Criterion Based on Threshold Value

When evaluating the validity of monitoring data of DC bias of transformers, it is not enough to evaluate the validity only based on the information of a single data point. Therefore, a certain data analysis period *S* needs to be selected. By analyzing the monitoring data, it was found that there were data points beyond the measuring range of the sensor in some time period. Since each sensor has its measuring range, if there are data points exceeding the range or equal to the boundary value in the monitoring data, it means that the data points of this fraction are abnormal data points. The threshold criterion based on the sensor measurement range is presented as follows.

**Criterion P_1_**: according to the measuring range of the corresponding sensor, set the upper limit *H*_0_ and lower limit *L*_0_ of the monitoring data.

This criterion is used to evaluate all data points in the selected time period *S*. If the value of data point *x_i_* exceeds the upper and lower limits of the measurement, that is, Equation (1) is satisfied, then the data point *x_i_* is judged to be an invalid data point.

(1) $$x_{i}\;\geq \;H_{0}\;or\;x_{i}\;\leq \;L_{0}.$$

According to the analysis of the characteristics of normal monitoring data under different working conditions in Section 2, it can be seen that whether the transformer is an auto-transformer or whether the transformer DC bias suppression device is turned on has a great impact on the characteristics of monitoring data. The threshold criterion based on the operating condition of the transformer is presented as follows.

**Criterion P_2_**: according to different transformer working conditions *Q*_1_–*Q*_3_, set effective upper limit *H*_1_–*H*_3_ and effective lower limit *L*_1_–*L*_3_ of monitoring data.

This criterion is used to evaluate all data points in the selected period S. First, the working condition *Q* of the transformer is determined, and then the data point *x_i_* is compared with the corresponding upper and lower limits of the monitoring data. If the upper and lower limits are exceeded, which satisfies Equation (2), then the data point *x_i_* is judged to be an invalid data point.

(2a) $$\begin{array}{c} Q_{1}:x_{i}\geq H_{1}\;or\;x_{i}\leq L_{1}, \end{array}$$

(2b) $$\begin{array}{c} Q_{2}:x_{i}\geq H_{2}\;or\;x_{i}\leq L_{2}, \end{array}$$

(2c) $$\begin{array}{c} Q_{3}:x_{i}\geq H_{3}\;or\;x_{i}\leq L_{3}. \end{array}$$

#### 3.1.2. Criterion Based on Data Continuity

According to the characteristics of the sensor and the time-varying characteristics of the measurement parameters, the continuous and identical data points directly reflect the fault of the sensors’ induction part or the system’s communication. Therefore, the continuous and identical data points are abnormal data points. The criteria based on continuous sameness of data are presented as follows.

**Criterion P3**: According to the tolerance of the number of continuously identical data, set the continuously identical data tolerance values *N*.

This criterion is used to evaluate all data points in the selected time period *S*. Starting from the initial data point *x*_1_, the continuous *N* data points are compared. If Equation (3) is satisfied, the data points *x_i_*, *x*_*i*+1_, ..., *x*_*i+n*-1_ are all invalid data points, and then compare the subsequent data points *x_i+N_*, *x*_*i+N*+1_, ... with *x_i_*. If they are the same as *x_i_*, they will be judged as invalid data points, until a data point different from *x_i_* appears or the last data point is judged.

(3) $$x_{i}=x_{i+1}=\ldots =x_{i+N-1}.$$

Due to the limited measurement accuracy of the sensor, it is impossible to identify small changes of monitoring data below its accuracy. Therefore, when the measured data are very small but not zero, a large number of continuous identical values will also appear in the neutral DC and vibration data. In order to avoid data validity misjudgment caused by this, the supplementary criterion P3 should be added.

**Supplementary criterion of criterion P_3_**: according to the range and measurement accuracy of the sensor, set the appropriate minimum applicable value *M*.

Using this supplementary criterion, if the value *x_i_* of the continuous identical data satisfies Equation (4), this part of data will not be judged as invalid data.

(4) $$x_{i}\;\neq \;0\;and\;\left|x_{i}\right|\;\leq \;M.$$

#### 3.1.3. Criterion Based on Impact Data

The peak value index is the quotient of the peak value and the effective value, and the pulse index is the quotient of the peak value and the mean value, both of which are used to judge whether there are impact data in the monitoring data. When the monitoring device is strongly interfered with by the outside, impact data will appear in the monitoring data, but this part of data has nothing to do with DC bias. Thus, criteria based on peak value and pulse value are proposed as follows:

**Criterion P_4_**: according to the tolerance degree of abnormal impact data, set the appropriate tolerance value *C* of the abnormal impact data.

For neutral DC data, the tolerance value *C*_1_ based on peak index was selected, while for noise and vibration data, the tolerance value *C*_2_ based on pulse index was selected. This criterion is used to evaluate all data points within the selected time period *S* of the corresponding type of data. First, according to Equations (5)–(7), the peak value *X_p_*, effective value *X_r_* or mean *X_av_* of the data in the period *S* can be obtained. Then, *I_p_* or *C_f_*, the peak value of data in period *S*, are calculated and compared with the corresponding abnormal impact tolerance value *C*. If Equation (8) or (9) are satisfied, abnormal impact data can be judged to exist in time period S.

(5) $$X_{p}=E[\max\left|x_{i}\right|],$$

(6) $$X_{r}=\sqrt{\frac{1}{n}\sum_{i=1}^{n}x_{i}^{2}},$$

(7) $$X_{av}=\frac{1}{n}\sum_{i=1}^{n}X_{i},$$

(8) $$I_{p}=\frac{X_{p}}{X_{r}}\;and\;I_{p}>C_{1},$$

(9) $$C_{f}=\frac{X_{p}}{X_{av}}\;and\;C_{f}>C_{2}.$$

Because the criterion is proposed from the perspective of statistical analysis, the criterion can only be used to judge whether there are abnormal impact data in time period S, rather than directly find abnormal data points. Therefore, it is necessary to supplement the criterion of data points analysis.

**Supplementary criterion of criterion P_4_**: according to the tolerance degree of data singularity, set the appropriate change rate *K* of data singularity tolerance and the corresponding change minimum index *K*’.

This criterion is used to evaluate all data points in the selected time period *S*. Starting from the second data point *x*_2_, data point *x_i_* is compared with the two before and after data points. If Equations (10)–(12) are satisfied at the same time, data point *x_i_* is judged to be an invalid data point. The purpose of setting the change minimum index is to avoid the miscalculation of data point singularity caused by a data point that is too small.

(10) $$\left(x_{i-1}-x_{i}\right)\left(x_{i+1}-x_{i}\right)>0,$$

(11) $$\left|\frac{x_{i-1}\;-\;x_{i}}{x_{i}}\right|>K\;and\;\left|\frac{x_{i+1}\;-\;x_{i}}{x_{i}}\right|>K,$$

(12) $$\left|x_{i-1}-x_{i}\right|>K^{\prime }\;and\;\left|x_{i+1}-x_{i}\right|>K^{\prime }.$$

#### 3.1.4. Criterion Based on Data Correlation

Transformer noise is mainly caused by transformer vibration; therefore, there is a strong correlation between noise intensity and vibration speed in monitoring data. If the noise intensity in the monitoring data changes greatly and the vibration velocity does not change correspondingly, then the noise data in this part of the change are not caused by the vibration, but by the background noise independent of the DC bias of the transformer. The criteria based on data correlation are presented as follows.

**Criterion P_5_**: according to the tolerance degree of data asynchronous changes, the appropriate data asynchronous tolerance rate *G* is set.

This criterion is used to make synchronous judgments on all noise intensity data points *x* and the vibration velocity data point *y* within the selected time period *S*. The rate of change is calculated from the initial data points *x*_1_ and *y*_1_. If the difference between the two rates of change satisfies Equation (13), the noise intensity data points *x_i_* and *x*_*i*+1_ are judged to be invalid data points.

(13) $$\left|\frac{x_{i+1}-x_{i}}{x_{i}}-\frac{y_{i+1}-y_{i}}{y_{i}}\right|>G.$$

### 3.2. Data Validity Evaluation Process

**Figure 11:**
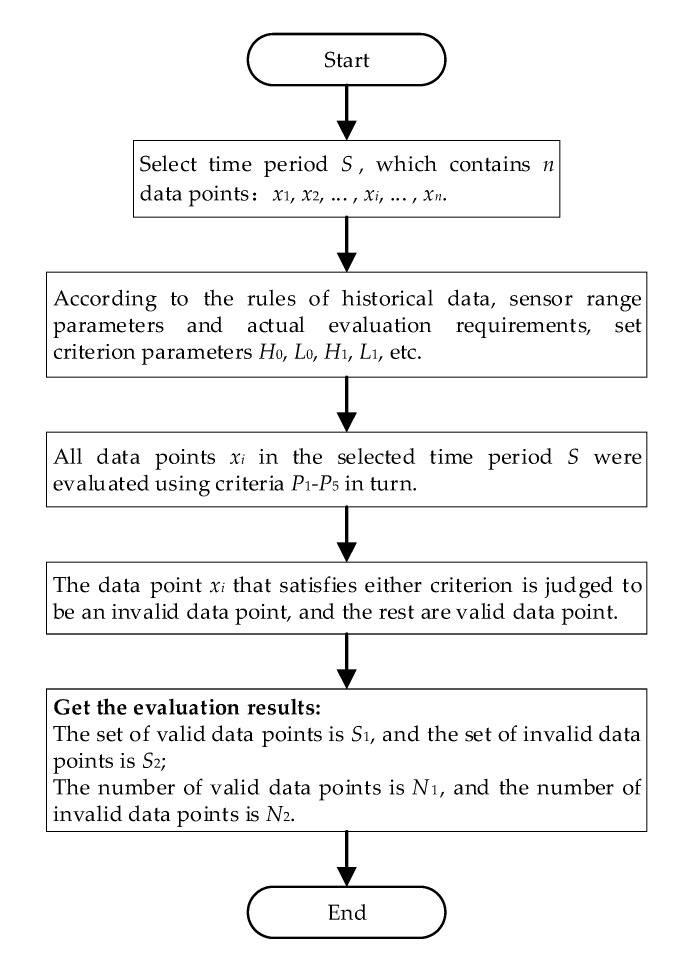
Data validity evaluation process.

The validity evaluation process of DC bias data includes four steps: selecting objects, setting criteria parameters, evaluating, and obtaining evaluation results. First of all, the monitoring data time period *S* to be analyzed should be selected. Time period *S* contains *n* data points. Then, according to the rules of historical data, sensor range parameters and actual evaluation requirements set criterion parameters *H*_0_, *L*_0_, *H*_1_, *L*_1_, etc. Next, each data point *x*_i_ in the selected time period *S* is evaluated by using the five criteria P_1_–P_5_ mentioned above. Each criterion reflects the validity of data point *x*_i_ in a certain aspect. As long as any criterion is satisfied, it is classified as an invalid data point, and the rest are valid data points. Finally, the data validity evaluation results are obtained, that is, the set of valid data points *S*_1_ and the set of invalid data points *S*_2_, the number of valid data points *N*_1_, and the number of invalid data points *N*_2_. The data validity evaluation process is shown in Figure 11.

According to statistical analysis, invalid data account for more than 10% when the monitoring device fails. Therefore, if the number of invalid data points exceeds 10% of the total number of data points in the evaluation results, the monitoring device of the transformer can be speculated to have a fault.

## 4. Case Studies and Results

In order to verify the validity of the proposed method in this paper, case studies were carried out on the data of the DC bias magnetic monitoring system of a regional power grid. Using this evaluation method, the validity of monitoring data was evaluated systematically and comprehensively. By manual reexamination, the judgment of invalid data was confirmed, and the validity of the method was verified.

### 4.1. Parameter Setting of Criterion

According to the main technical parameters of the measurement sensor, the parameters of data validity evaluation Criterion 1 were set. According to the statistical rules of DC bias monitoring data obtained by many tests and the general requirements for the DC bias parameters of the transformer, the parameters of data validity evaluation Criterion 2–5 were set. The specific parameter setting of data validity evaluation criteria are shown in Table 3.

### 4.2. Case Studies

#### 4.2.1. Case 1: Data Validity Evaluation of Transformer C

**Figure 12:**
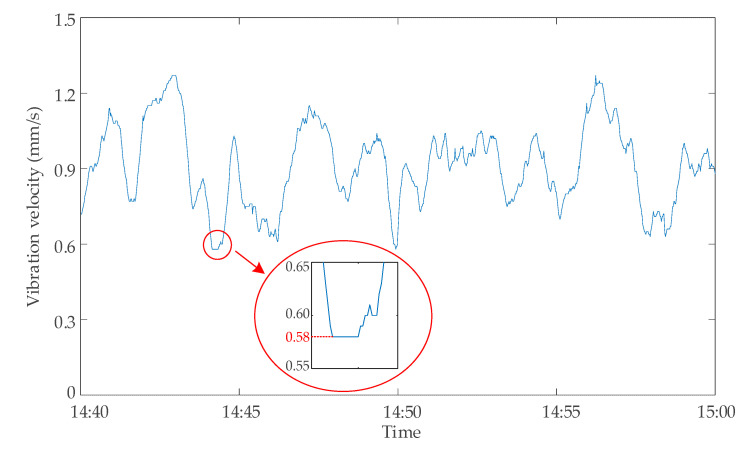
Monitoring data of vibration velocity of transformer C on 28 April 2020.

The method proposed in this paper was used to evaluate the data validity of the transformer C in the regional power grid. There was a period of continuous identical data lasting for 12 s from 14:40 to 15:00 on 28 April 2020. The vibration velocity monitoring data of the transformer from 14:44:08 to 14:44:19 were 0.58 mm/s, as shown in Figure 12. According to the setting of the continuous same tolerance value *N* in Criterion 3, the continuous same number was greater than the tolerance value *N* = 10. Therefore, the data points of this part were judged as invalid data points. After querying the network communication records of the monitoring system, it was found that a temporary data communication failure occurred during this period, which led to the abnormal situation shown in Figure 12. Through the analysis of this case, it can be seen that the validity evaluation method of the DC bias data proposed in this paper can well identify the data anomalies that are difficult to observe artificially.

#### 4.2.2. Case 2: Data Validity Evaluation of Transformer D

**Figure 13:**
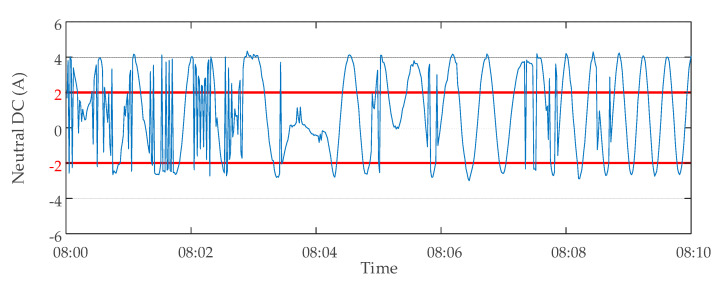
Monitoring data of neutral DC of transformer D on 30 October 2019.

**Figure 14:**
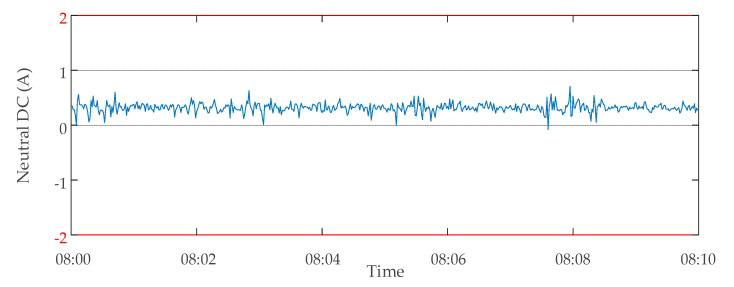
Monitoring data of neutral DC of transformer D on 05 November 2019.

The method proposed in this paper was used to evaluate the data validity of the transformer D. The monitoring data of transformer D were abnormal on 30 October 2019. The monitoring data of the neutral point DC at 8:00–8:10 of the transformer are shown in Figure 13. When the transformer was a non-autotransformer and the suppression device was on (working condition *Q*_2_), a large amount of neutral DC data were judged invalid because they exceeded the upper limit *H*_2_ (2A) or the lower limit *L*_2_ (−2A) of Criterion 2. Through the analysis of these invalid data, it was found that the cause of this part of invalid data could be attributed to the abnormal zero drift of data. The monitoring device was presumed to have malfunctioned because of a large amount of invalid data. The inspection of the neutral point DC monitoring device of the transformer showed that there was indeed a serious fault. After the device was replaced on 4 November 2019, the monitoring data of the same period were analyzed again the next day using the same method. The results showed that there were no longer many invalid data, as shown in Figure 14. The results of two data validity analyses of transformer D are shown in Table 4.

#### 4.2.3. Case 3: Data Validity Evaluation of Transformer E

**Figure 15:**
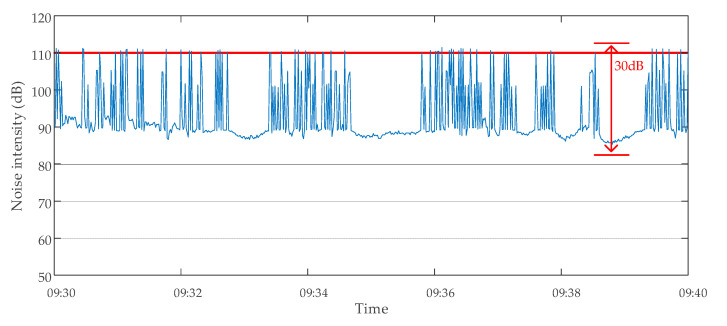
Monitoring data of noise intensity of transformer E on 18 October 2019.

**Figure 16:**
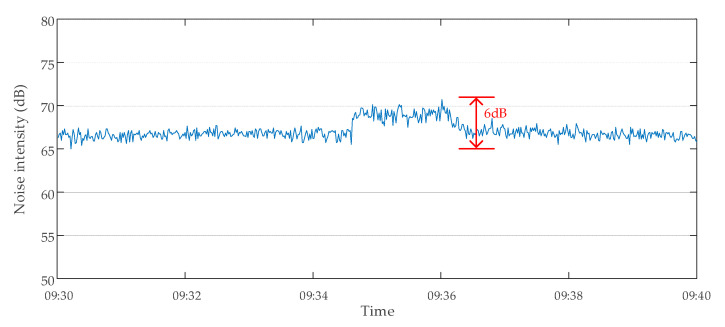
Monitoring data of noise intensity of transformer E on 5 November 2019.

The method proposed in this paper was used to evaluate the data validity of the transformer E. The monitoring data of transformer E were abnormal on 18 October 2019. The monitoring data of transformer noise intensity at 9:30–9:40 are shown in Figure 15. A large number of intermittent continuous anomalous hopping data occurred in the noise intensity data. These data were judged invalid because they exceeded the upper limit *H*_1_ (110 dB) in Criterion 2 and the data singularity tolerance value (10%) in Criterion 4. The monitoring device was presumed to have malfunctioned because of a large amount of invalid data. The inspection of the noise monitoring device of the transformer showed that there was indeed a serious fault. After the device was replaced on 4 November 2019, the monitoring data of the same period were analyzed again the next day using the same method. The results showed that there were no longer many invalid data, as shown in Figure 16. The results of two data validity analyses of transformer E are shown in Table 5.

## 5. Conclusions

This paper proposes a validity evaluation method based on data driving for on-line monitoring data of the transformer under DC-bias. First, the variation rule and threshold range of monitoring data for neutral point DC, vibration, and noise of the transformer under different working conditions are obtained through statistical analysis. Then, according to the characteristics of normal and abnormal data, the data validity criteria based on data threshold, continuity, impact, and correlation are proposed in this paper. Using these criteria, a comprehensive evaluation system for data validity of DC bias is established.

The method proposed in this paper is used to evaluate the validity of the real measured data of the DC bias magnetic monitoring system of a regional power grid. The results show that it can replace the traditional manual method to evaluate data validity. In addition, when there is a large amount of invalid data in the evaluation results, it can be inferred that the monitoring device fails, and the correctness of the fault judgment of the monitoring device can be confirmed through manual reexamination.

One insufficiency of this paper lies in the classification of working conditions. In this paper, the working conditions are classified only from the three perspectives of DC bias generation, suppression device input, and autotransformer. The influence of voltage class, saturation degree, and power factor of the transformer is ignored. In our future work, we will strive to find a better data classification method.

All in all, the data validity evaluation method proposed in this paper can systematically and comprehensively evaluate the validity of the DC bias monitoring data, laying a foundation for the subsequent analysis of DC bias characteristics.

## Figures and Tables

**Table 1:** Characteristic rules of normal data.

Transformer Type	Rated Power (MVA)	Rated Voltage (kV)
ODFPSZ9-250000/525/√3 SFSZ9-240000/220	250/250/53.5 240/240/80	525/√3/242/√3/34.5 220/115/10.5
SFSZ9-180000/220	180/180/0	220/121/11
SFSZ9-150000/220	150/150/75	220/121/11

**Table 2:** Characteristic rules of normal data.

Working Condition	Neutral DC	Noise Intensity	Vibration Velocity
*Q*_1_	−90–90 A	50–110 dB	0–2 mm/s
*Q*_2_	−2–2 A	50–90 dB	0–0.7 mm/s
*Q*_3_	−2–2 A	50–110 dB	0–2 mm/s

**Table 3:** Parameter setting of data validity evaluation criterion.

Criterion Parameter		Neutral DC	Noise Intensity	Vibration Velocity
Criterion 1	*H*_0_	100 A	130 dB	20 mm/s
	*L*_0_	−100 A	30 dB	0 mm/s
Criterion 2	*H*_1_	90 A	110 dB	2 mm/s
	*L*_1_	−90 A	50 dB	0 mm/s
	*H*_2_	2 A	90 dB	0.7 mm/s
	*L*_2_	−2 A	50 dB	0 mm/s
	*H*_3_	2 A	110 dB	2 mm/s
	*L*_3_	−2 A	50 dB	0 mm/s
Criterion 3	*N*	10	10	10
	*M*	0.3 A	\	0.15 mm/s
Criterion 4	*C*	1.5	1.1	1.1
	*K*	20%	10%	10%
	*K’*	5 A	5 dB	0.2 mm/s
Criterion 5	*G*	\	10%	

**Table 4:** Transformer D data validity analysis results.

Date of Analysis	Time Period of Analysis	Total Number of Data Points	Number of Normal Data Points	Number of Invalid Data Points
30 October 2019	08:00–08:10	600	252	348
5 November 2019	08:00–08:10	600	600	0

**Table 5:** Transformer E data validity analysis results.

Date of Analysis	Time Period of Analysis	Total Number of Data Points	Number of Normal Data Points	Number of Invalid Data Points
18 October 2019	09:30–09:40	600	504	96
5 November 2019	09:30–09:40	600	600	0

## References

[1] Yuan P., Mao W., Ye H., Liu Y. Model Construction and Analysis of Transformer DC Magnetic Bias Induced by Rail Transit Stray Current. Proceedings of the 2019 IEEE 3rd Conference on Energy Internet and Energy System Integration.

[2] Ni Y.-R., Zeng X.-J., Yu K., Leng Y., Peng P. Research on Modeling Method of Transformer DC Bias Caused by Metro Stray Current. Proceedings of the 2018 International Conference on Power System Technology.

[3] Lin S., Zhou Q., Lin X.-H., Liu M.-J., Wang A.-M. (2020). Infinitesimal method based calculation of metro stray current in multiple power supply sections. IEEE Access.

[4] He J.-L., Yu Z.-Q., Rong Z., Zhang B. (2012). Vibration and audible noise characteristics of AC transformer caused by HVDC system under monopole operation. IEEE Trans. Power Del..

[5] Girgis R., Vedante K. Effects of GIC on power Transformers and Power Systems. Proceedings of the IEEE Transmission and Distribution Conference and Exposition.

[6] Jiang W., He L., Zhang Z.-X. Monitoring and Suppression Measures of Transformer DC Bias Current. Proceedings of the International Conference on Condition Monitoring and Diagnosis.

[7] Wang A.-M., Lin S., Hu Z.-H., Li J.-Y., Wang F., Wu G.-X., He Z.-Y. (2020). Evaluation model of DC current distribution in AC power systems caused by stray current of DC metro systems. IEEE Trans. Power Del..

[8] Wu T.-Y., Chen L. Data Mining Based on DC Bias On-Line Monitoring System of Shanghai Power Grid. Proceedings of the IEEE Conference on Energy Internet and Energy System Integration.

[9] Liu B.-W., Ma H., Zhou L.-X. On-line Monitoring of Transformer Vibration and Noise Based on DC Magnetic Bias. Proceedings of the International Conference on Intelligent Systems Design and Engineering Applications.

[10] Cui M.-D., Yang Q., Liu L.-G. Application of the Virtual Instrument Technology in the Monitoring System of Transformer DC Magnetic Bias. Proceedings of the International Conference on Computer Application and System Modeling.

[11] Liu Y.-Q., Lu J.-Y., Zhang Q., Guo J. (2014). Effectiveness determination method of audible noise test data for high voltage dc transmission lines. High Volt. Eng..

[12] Jia J., Tao F.-B., Zhang G.-J., Shao J., Zhang X.-H., Wang B. (2020). Validity evaluation of transformer DGA online monitoring data in grid edge systems. IEEE Access.

[13] Qiao J., Liu Q., Zhang Y.-F. Design of Geomagnetic Induction Current Monitoring and Early Warning System Based on Cloud Server. Proceedings of the IEEE Conference on Industrial Electronics and Applications.

[14] Liu C., Zhou X.-X., Tian H.-Y., Zhao Y., Qu T.-H., Chen W.-Z. Research on DC BIAS Current Monitoring of Power Transformer Neutral Point. Proceedings of the IEEE International Conference on High Voltage Engineering and Application.

[15] Tong X., Quan J.-T., Xia T., He J.-H., Wang C.-Z. Design and Application of Sensor Measuring Neutral DC Current of Transformer Based on Resistance Sampling. Proceedings of the IEEE Sustainable Power and Energy Conference.

[16] Xu S.-W., Su D.-H., Wang X. The Data Validity Evaluation in Land Change Survey Based on Remote Sensing. Proceedings of the International Conference on Geoinformatics.

[17] Zhou N.-N., Huang G.-F., Zhong S.-Y. (2018). Big data validity evaluation based on MMTD. Math. Probl. Eng..

[18] Xie C.-C., Shao M.-H. (2018). An evaluation on integrity and validity of traffic data collected from urban expressways. J. Trans. Inf. Saf..

[19] Liu S., Zhao Y., Lin Z., Ding Y., Yan Y., Yang L., Wang Q., Zhou H., Wu H. (2019). Data-driven condition monitoring of data acquisition for consumers’ transformers in actual distribution systems using t-statistics. IEEE Trans. Power Del..

[20] Ijaz M.F., Attique M., Son Y. (2020). Data-driven cervical cancer prediction model with outlier detection and over-sampling methods. Sensors.

[21] Turrin S., Subbiah S., Leone G., Cristaldi L. An Algorithm for Data-Driven Prognostics Based on Statistical Analysis of Condition Monitoring Data on a Fleet Level. Proceedings of the 2015 IEEE International Instrumentation and Measurement Technology Conference (I2MTC).

[22] Yin S., Ding S.X., Xie X., Luo H. (2014). A review on basic data-driven approaches for industrial process monitoring. IEEE Trans. Ind. Electron..

[23] Jayaraman B., Mamun S.M.A.A. (2020). On data-driven sparse sensing and linear estimation of fluid flows. Sensors.

[24] Azimi M., Eslamlou A.D., Pekcan G. (2020). Data-driven structural health monitoring and damage detection through deep learning: State-of-the-art review. Sensors.

[25] Xie Z.-C., Lin X.-N., Zhang Z.-Y., Li Z., Xiong W., Hu H., Khalid M.S., Adio O.S. (2017). Advanced DC bias suppression strategy based on finite dc blocking devices. IEEE Trans. Power Del..

[26] Zeng R., Yu Z.-Q., He J.-L., Zhang B., Niu B. (2011). Study on restraining DC neutral current of transformer during HVDC monopolar operation. IEEE Trans. Power Del..

[27] Bolduc L., Granger M., Pare G., Saintonge J., Brophy L. (2005). Development of a DC current-blocking device for transformer neutrals. IEEE Trans. Power Del..

[28] Wu G.-X., Chen L., Shi Y.-T., Lin S. (2020). Analysis on test and suppression of DC bias of 500 kV main transformer in shenzhen substation. Guangdong Electr. Power.

[29] Wu H., Wang H.-B., Zhang P., Huang M., Qi B., Rong Z.-H. The Assess Method of Validity for Partial Discharge Sensor Based on Multiple Criterion. Proceedings of the IEEE Conference on Electrical Insulation and Dielectric Phenomena.

[30] Zhou Z.-Q., Xiao L., Nie D.-X., Qi B., Zhang P., Gao C.-J., Li C.-R. Validity Evaluation Method of DGA Monitoring Sensor in Power Transformer Based on Chaos Theory. Proceedings of the IEEE Conference on Electrical Insulation and Dielectric Phenomena.

[31] Zhang P., Qi B., Rong Z.-H., Li C.-R., Li F. The Assess Method of Validity for DGA Sensor Based on Multiple Criterion. Proceedings of the IEEE Electrical Insulation Conference.

